# A prognostic model for anoikis-related genes in pancreatic cancer

**DOI:** 10.1038/s41598-024-65981-7

**Published:** 2024-07-02

**Authors:** Wenbin Song, Haiyang Hu, Zhengbo Yuan, Hao Yao

**Affiliations:** 1 Department of General Surgery, Tianjin Medical University General Hospital, Tianjin, 300052 People’s Republic of China; 2 Tianjin Key Laboratory of Precise Vascular Reconstruction and Organ Function Repair, Tianjin, 300052 People’s Republic of China; 3 Department of Cardiac Critical Care Medicine, Affiliated Hospital of Jining Medical University, Jining, 272007 People’s Republic of China; 4 School of Medicine, Xiamen University, No.4221 Xiangan South Road, Xiangan District, Xiamen, 361102 People’s Republic of China; 5 Department of Neurosurgery, The First Affiliated Hospital of Xiamen University, School of Medicine, Xiamen University, No.55 Zhenghai load, Siming District, Xiamen, 361001 People’s Republic of China; 6 Department of Hepatological Surgery, The Second Hospital of Tianjin Medical University, No.23 Pingjiang Road, Hexi District, Tianjin, 300211 People’s Republic of China

**Keywords:** Pancreatic cancer, Anoikis, Prognostic model, TCGA-PAAD, ICGC-PACA, Computational biology and bioinformatics, Gastroenterology, Medical research, Molecular medicine, Risk factors

## Abstract

Anoikis, a distinct form of programmed cell death, is crucial for both organismal development and maintaining tissue equilibrium. Its role extends to the proliferation and progression of cancer cells. This study aimed to establish an anoikis-related prognostic model to predict the prognosis of pancreatic cancer (PC) patients. Gene expression data and patient clinical profiles were sourced from The Cancer Genome Atlas (TCGA-PAAD: Pancreatic Adenocarcinoma) and the International Cancer Genome Consortium (ICGC-PACA: Pancreatic Ductal Adenocarcinoma). Non-cancerous pancreatic tissue gene expression data were obtained from the Genotype-Tissue Expression (GTEx) project. The R package was used to construct anoikis-related PC prognostic models, which were later validated with the ICGC-PACA database. Survival analyses demonstrated a poorer prognosis for patients in the high-risk group, consistent across both TCGA-PAAD and ICGC-PACA datasets. A nomogram was designed as a predictive tool to estimate patient mortality. The study also analyzed tumor mutations and immune infiltration across various risk groups, uncovering notable differences in tumor mutation patterns and immune landscapes between high- and low-risk groups. In conclusion, this research successfully developed a prognostic model centered on anoikis-related genes, offering a novel tool for predicting the clinical trajectory of PC patients.

## Introduction

PC is a leading cause of cancer-related death globally, ranking as the seventh most common cause of cancer fatalities worldwide^1^. Approximately 95% of PC are exocrine cell tumors, with the most prevalent type being pancreatic ductal adenocarcinoma^2^. There are currently no reliable screening techniques for pancreatic ductal adenocarcinoma, and a significant percentage of patients (30–35%) are diagnosed with advanced or metastatic disease (50–55%), contributing to the high mortality rate associated with this type of cancer^3^. In the span of the last 27 years, the occurrence rate of PC has nearly doubled.The aging population and exposure to risk factors like smoking, obesity, diabetes, and alcohol consumption are expected to contribute to a further rise in PC cases in the coming decades, with a shift towards affecting younger individuals^4^.

The emergence and development of immunotherapy and targeted therapy strategies have greatly changed the prognosis of many patients with malignant tumors, improving survival rates to varying degrees. Although immunotherapy and targeted therapy have been successful in the treatment of many solid tumors in the past decades, these drugs have not shown significant benefits for PC patients^5^. Therefore, chemotherapy is still one of the few effective treatment strategies for advanced PC. Summarizing the existing research results, it is shown that: immunotherapy and targeted therapy did not yield practice-changing results in pancreatic cancer, probably because PC tumor microenvironment (TME) and tumor immune microenvironment (TIME) are peculiar compared to most tumors, as is the genomic landscape that accompanies this disease^6^. PC has a unique tumor microenvironment that contributes to its resistance to various treatments. This environment is characterized by dense and heterogeneous stroma, primarily composed of cells with diverse functions, including fibroblasts, myofibroblasts, immune cells^7^. These components collectively promote tumor growth and metastasis and create high hydrostatic pressure within tumor vessels, which can limit cell trafficking, particularly of immune cells^8^. Therefore, targeting the tumor microenvironment may be an effective strategy to overcome drug resistance and enhance immune cell infiltration in PC. Therefore, more research work should be started on efficient TME modification mechanisms that could make the tumor more immunosensitive. Whatever disease situation, accept or immunotherapy and targeted therapy can predict the clinical outcome of patients with pancreatic cancer biomarkers is crucial.

The population of PC patients with high microsatellite instability/mismatch repair deficiency is currently the only population that may benefit from immunotherapy; nevertheless, the prevalence of these alterations is too low to determine a real change in the treatment scenario of this tumor^6^. Current research shows that the number of cells expressing PD-1 and PD-L1 is lower in PC compared with tumors where immunotherapy demonstrated an established efficacy, such as malignant melanoma^9^. In addition, previous studies have confirmed that: CD40 activation may represent a strategy to reverse T-cell exhaustion, enhancing the anti-cancer effects of the TIME. Consistently, agonistic CD40 antibodies were shown to increase T-cell mediated cancer death and, in combination with chemotherapy, may rescue ICI sensitivity^10–13^. Therefore, the combination of single-agent ICI or a dual immune blockade has been considered to be a feasible therapeutic regimen in the standard regimen of existing chemotherapy, but subsequent studies have shown that it has no definite significance for the survival improvement of patients with PC^14,15^.

Among vaccination strategies, oncolytic viruses have been extensively researched in PC, either alone or in combination with conventional therapies. Encouraging results have been obtained in preclinical and clinical studies evaluating the potential of vaccinia virus, reovirus, herpes simplex virus-1, and adenovirus as oncolytic viruses. However, the dense and low-penetrability nature of pancreatic cancer TME limits the access of these viruses^16^. Transferring knowledge about the effectiveness of immunotherapy and targeted therapy from other types of cancer to pancreatic cancer is not enough to achieve promising results. It requires a deeper understanding of TME cells and their interactions. Further research is essential to better identify patients who may benefit from immunotherapy and to develop effective TME modification strategies to make the tumor more responsive to immunotherapy^6^.

Additionally, there have been encouraging results regarding personalized treatment approaches for PC. The tropomyosin receptor kinase (TRK) family has recently become a focal point in research. TRK comprises three transmembrane protein receptors: TrkA, TrkB, and TrkC, which are encoded by the NTRK1, NTRK2, and NTRK3 genes, respectively. These receptors regulate numerous aspects of neuronal development and function^17,18^. Chromosomal translocations involving NTRK1/2/3 genes result in constitutive activation and aberrant expression of TRK kinases^19^. NTRK alterations are infrequently found in tumors with high prevalence, such as PC, occurring in less than 1% of patients^20^. Recently, specific targeted therapies for NTRK-fusion positive tumors have emerged. In November 2018, the Food and Drug Administration approved larotrectinib for the treatment of adult and pediatric patients with NTRK-fusion positive tumors, which are either without a known acquired resistance mutation, metastatic, or in which surgical resection is likely to result in severe morbidity, and without effective alternative treatments or those that have progressed following treatment. Although this treatment approach is considered a form of palliative care in practical applications^21^.

Anoikis is a form of programmed cell death that occurs when specific epithelial cells lose attachment from their original site, activating the anoikis pathway to prevent them from attaching elsewhere. However, certain non-adherent cells, such as leukocytes, can bind to extracellular matrix (ECM) proteins, activating an inhibitory pathway that blocks anoikis. Cancer cells, resistant to anoikis, do not require ECM protein binding to avoid the induction of cell death signals^22^. Up to now, a multitude of research has shown that the lack of programmed cell death in cell clusters plays a pivotal role in both tumor growth and metastasis^23,24^. Like many other forms of cancer, PC cells are able to survive because they have developed resistance to a process called anoikis. Hence, focusing on the diminished resistance to apoptosis may serve as a viable approach in the therapeutic treatment of PC^25^.

## Materials and methods

### Data download

In the pursuit of creating precise prognostic models for predicting outcomes in PC patients, this research utilized 348 samples from worldwide databases. The transcriptomic data and associated clinical details of these samples (Table 1) were derived from The Cancer Genome Atlas (TCGA) and Genotype-Tissue Expression (GTEx) databases, comprising 177 cancerous tissue samples and 171 normal tissue samples. To broaden the model’s relevance, additional PC sample data, excluding those with incomplete clinical information, were gathered from the International Cancer Genome Consortium (ICGC). Anoikis-related genes (ARGs) were identified through a search for “Anoikis” on the GeneCards website. It is important to note that the study omitted samples from the TCGA database with a total survival time of under 30 days to reduce the influence of external variables, such as complications following surgery.

**Table 1:** Clinical information for theTCGA and ICGC cohorts.

Clinical feature	Type	TCGA		ICGC	
		Number	%	Number	%
Total		156	100	137	100
Age(years)	≤ 65	75	48	72	53
	> 65	81	52	65	47
Gender	Female	73	47	63	46
	Male	83	53	74	54
Tumor grade	G1	19	12	/	/
	G2	90	58	/	/
	G3	46	29	/	/
	G4	0	0	/	/
	Gx	1	1	/	/
Tumor stage	I	13	8	49	36
	II	134	86	81	59
	III	3	2	5	4
	IV	4	3	0	0
	Unknown	2	1	1	1
Tumor classification	T1	6	4	/	/
	T2	16	10	/	/
	T3	130	83	/	/
	T4	3	2	/	/
	Tx	1	1	/	/
Node classification	N0	42	27	/	/
	N1	112	72	/	/
	N2	0	0	/	/
	N3	0	0	/	/
	Unknown	2	1	/	/
Metastasis classification	M0	73	47	/	/
	M1	4	3	/	/
	Unknown	79	50	/	/

### Data information processing

The limma package within R was employed to juxtapose data from the TCGA and GTEx databases. The criteria for this analysis were set as log2FC greater than 1.0, an FDR below 0.05, and a P-value under 0.05. Subsequently, the identified potential target genes were cross-referenced with the ICGC database to pinpoint the final set of differentially expressed genes (DEGs). Additionally, to enhance the accuracy and reliability of the prognostic model, the TCGA dataset was refined by removing outliers using the WGCNA package.

Identification of differentially expressed anoikis -related genes.

Anoikis-related genes were acquired from the GeneCards website and then filtered based on specific criteria (relevance score > 1.5, GIFTs score > 15). The selected genes were compared with the differentially expressed genes (DEGs) to identify those specifically associated with anoikis and exhibiting differential expression. These DEGs were further analyzed for enrichment using GO and KEGG pathways. The identified genes will undergo additional analysis to determine their differential expression and prognostic significance in relation to anoikis.

### Construction and validation of anoikis-related gene risk signature

We utilized PC patient samples from the TCGA-PAAD database (n = 156) as the training set, and samples from ICGC-PACA (n = 137) as the validation set. In the TCGA-PAAD cohort, a univariate Cox hazard regression analysis was conducted to identify the genes linked to survival rates.Subsequently, to avoid overfitting, we used lasso regression to select genes (*p* < 0.05). The genes identified through lasso regression were further refined using multifactorial cox regression to identify the most pertinent ARGs associated with overall survival (OS). The formula for computing the risk score is delineated as follows:

$${\text{risk}}\;{\text{score}} = \sum\nolimits_{k = 1}^{n} {{\text{coef}}\left( {{\text{ARG}}^{{\text{k}}} } \right) \times {\text{expr}}\left( {{\text{ARG}}^{{\text{k}}} } \right)}$$

The term “coef” stands for coefficient, and expr(ARGk) represents the gene expression levels, which is used to get the risk score for each sample. Within the TCGA group, patients were categorized into either high-risk or low-risk categories, determined by the median value of the risk scores. Moreover, the efficacy of the model was evaluated by dividing tumor samples from the ICGC cohort into high and low risk subgroups, applying the identical formula.

### Risk score-related survival analysis and clinical characterization

We created survival analysis curves, survival status plots, and risk curves using risk scores for both high- and low-risk subgroups in the TCGA cohort of 156 samples. For model validation, the R package was employed to create the same curves for 137 samples within the ICGC cohort. Time-dependent ROC curves for 1, 2, and 3 years were also plotted for both cohorts. Additionally, univariate and multivariate Cox regression analyses were performed on clinical variables, including age, gender, tumor grade, tumor stage, and risk score, using data from 156 samples in the TCGA cohort. ROC curves for clinical characteristics and risk scores were also plotted. Finally, we developed nomograms to predict the likelihood of a patient’s clinical outcome.

### Tumor mutation load analysis

Information regarding tumor mutations in PC patients was sourced from the TCGA database, and visualization of this data was accomplished using the maftools package. Initially, the mutation status and correlations of mutant genes in the TCGA cohort were examined. Subsequently, waterfall plots of the top 10 mutant genes for all samples in the TCGA cohort were generated. To explore the association between the risk score and the frequency of tumor mutations, a detailed analysis of the mutant gene waterfall plots was conducted for both high-risk and low-risk groups. The top 10 mutated genes were specifically chosen for the waterfall plots of both subgroups. Moreover, tumor mutation burden (TMB) scores were calculated for all TCGA samples. After excluding hypermutated samples, the remaining ones were segregated into high TMB (H-TMB) and low TMB (L-TMB) groups, using the median TMB values as the dividing criterion. The impact of both risk characteristics and TMB values on patient prognosis was then assessed. Kaplan–Meier survival curves were constructed for the H-TMB and L-TMB groups, and subsequently for the combination of high and low risk and high and low TMB values.

### Influence of genes associated with anoikis on the immune microenvironment of tumors

Utilizing the gene expression data from tumor samples, the abundance of immune cells within these samples was quantified using the CIBERSORT package. A survival analysis, in conjunction with the infiltration of immune cells, was conducted to investigate their correlation with tumor prognosis. Subsequently, the ESTIMATE package was applied to determine the immune scores of the samples, and violin plots were created to juxtapose these scores between the high-risk and low-risk groups. For assessing the potential effectiveness of immunotherapy, TIDE scores were generated via the website (http://tide.dfci.harvard.edu/) to examine the variance in these scores across the high-risk and low-risk categories.

## Result

### Identification of anoikis-related PC differentially expressed genes

**Figure 1:**
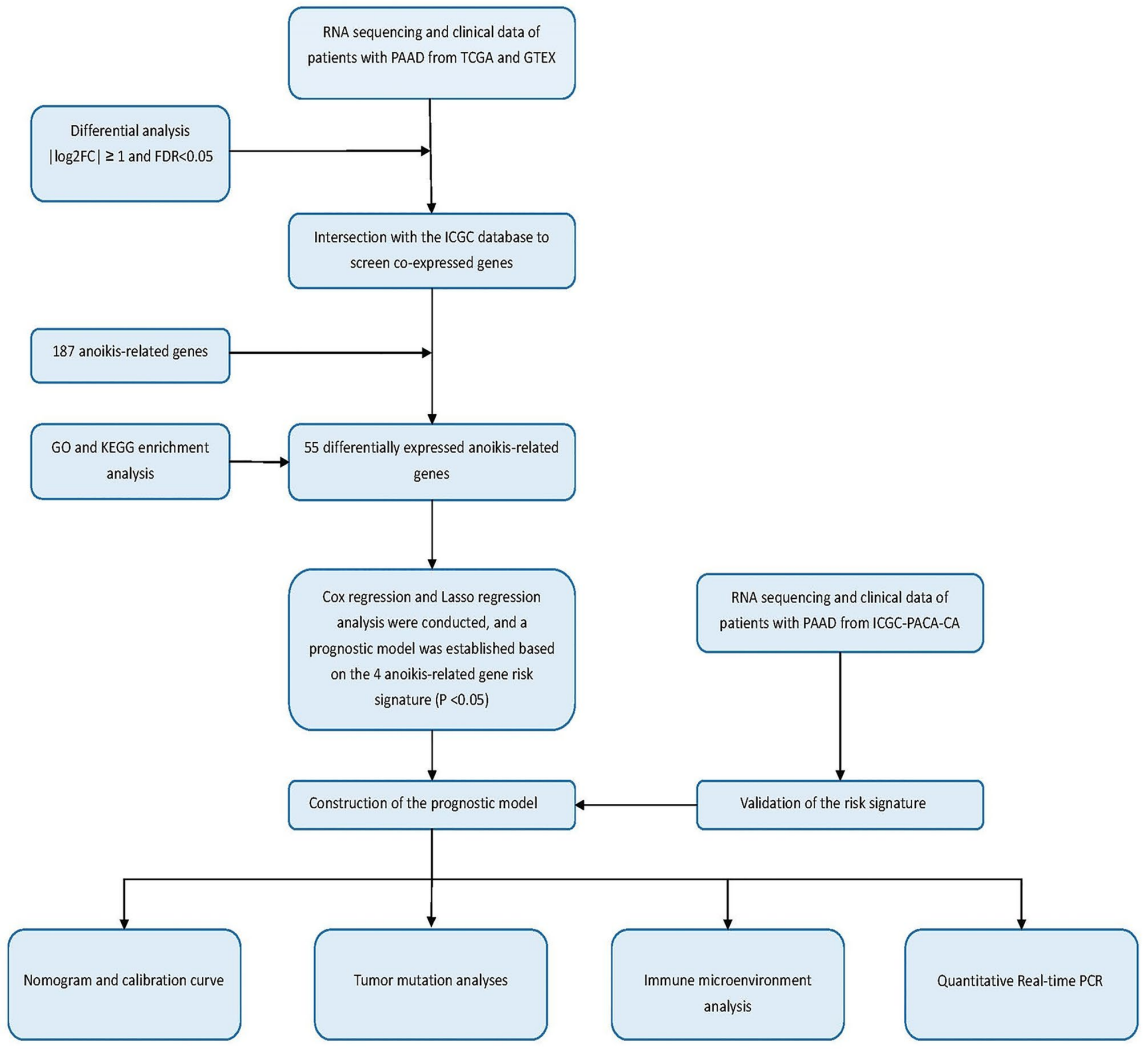
Flowchart of this study.

**Figure 2:**
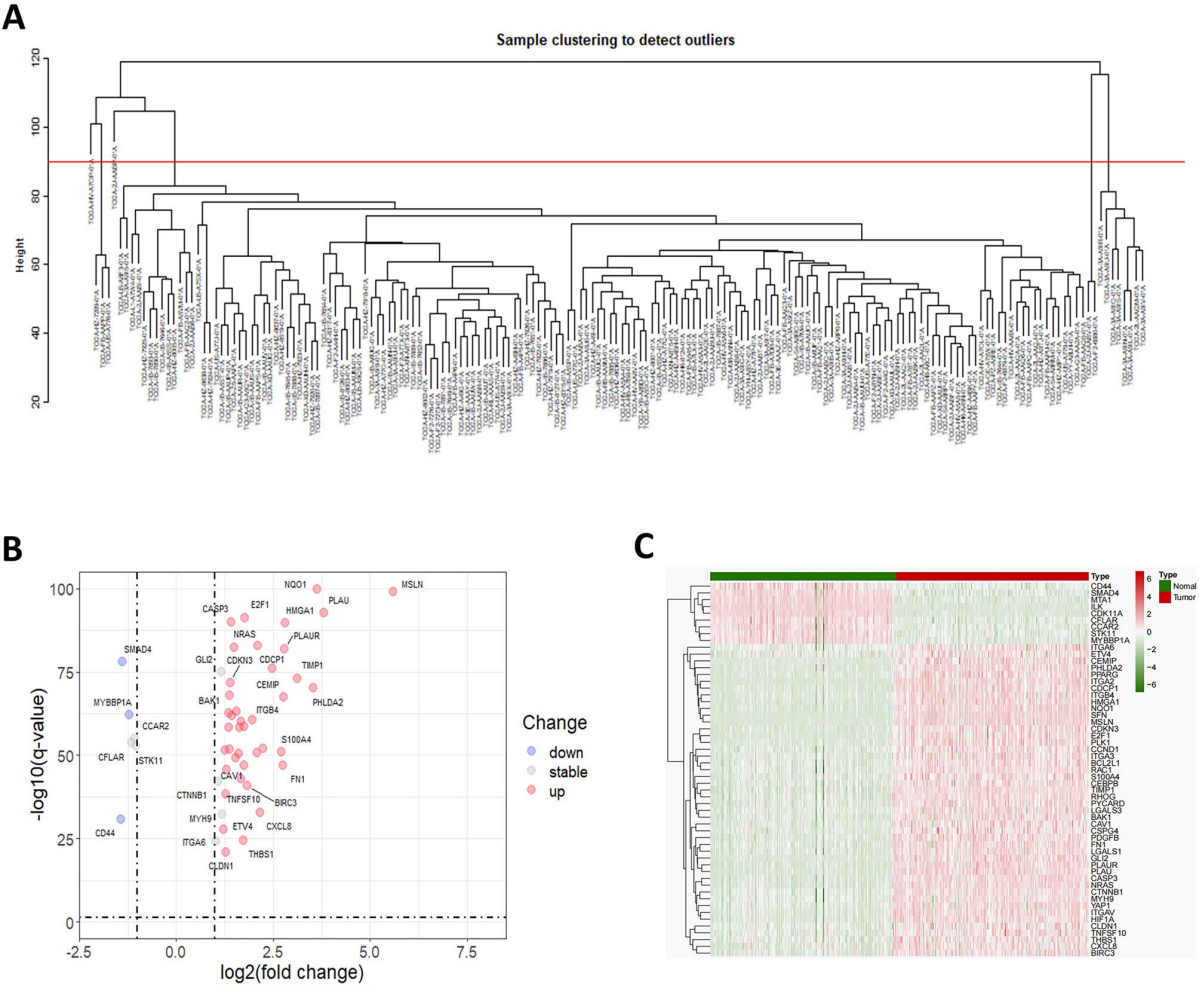
Identification of differentially expressed genes associated with loss of nest apoptosis. (**A**) TGCA cohort sample analysis, each sample corresponds to a dendrite and the red line is the exclusion of outlier sample bounds. (**B**) Volcano plot of loss-of-nest apoptotic genes, red represents highly expressed genes and blue the opposite. (**C**) Correlation heat map showing differential expression of target genes in tumor and normal samples.

The flowchart of this study is depicted in Fig. 1. The WGCNA package was applied to remove outliers from the TCGA tumor samples (Fig. 2A). The limma package in the R environment was used for differential expression analysis of genes in both tumor and normal samples from the TCGA and GTEx databases, identifying a total of 5952 differentially expressed genes. Subsequently, 2537 genes were identified by overlapping with the expressed genes from 137 samples in the ICGC. The intersection of these genes with the 187 identified anoikis-related genes yielded 55 anoikis differentially expressed genes linked to PC prognosis. Volcano plots were generated based on these 55 genes and annotated with gene names (Fig. 2B). The heatmap showed that these 55 genes exhibited varying levels of expression in tumor and normal samples (Fig. 2C).

### GO and KEGG enrichment analysis

**Figure 3:**
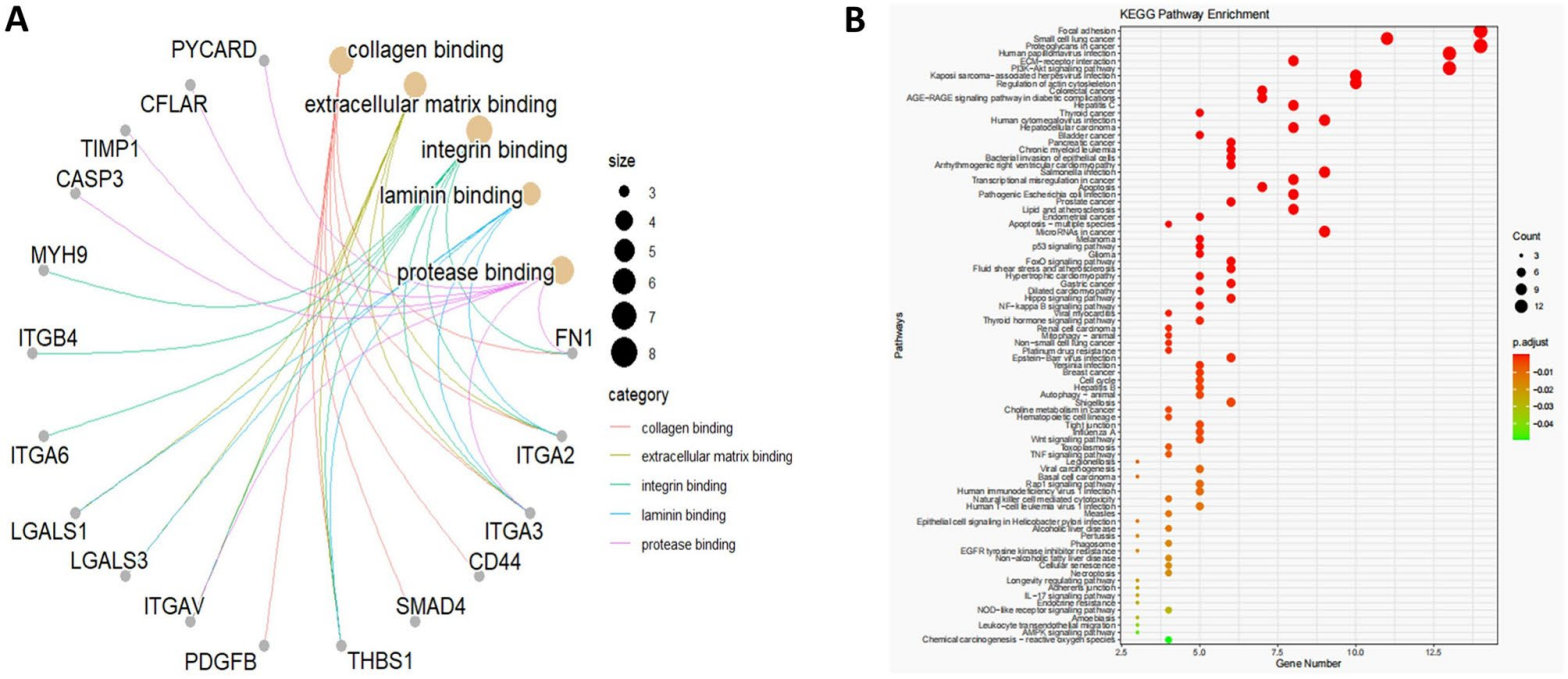
GO and KEGG enrichment analysis of target genes. (**A**) Five related pathways that were mainly clustered after GO enrichment analysis of the target genes. (**B**) KEGG enrichment analysis shows that most of the target genes are clustered in pathways related to tumorigenesis and cell adhesion.

Examination of genes tied to the prognosis of PC and their relevance to anoikis revealed that most were predominantly involved in activities related to collagen binding, extracellular matrix interaction, and integrin binding. These activities are generally associated with processes such as cell adhesion, movement, cell death, and inflammatory responses (Fig. 3A). Furthermore, pathway enrichment analysis using KEGG revealed that a significant number of these genes were categorized within pathways linked to cancer progression and cellular adhesion (Fig. 3B).

### Modeling of risk signature of anoikis-related genes

**Figure 4:**
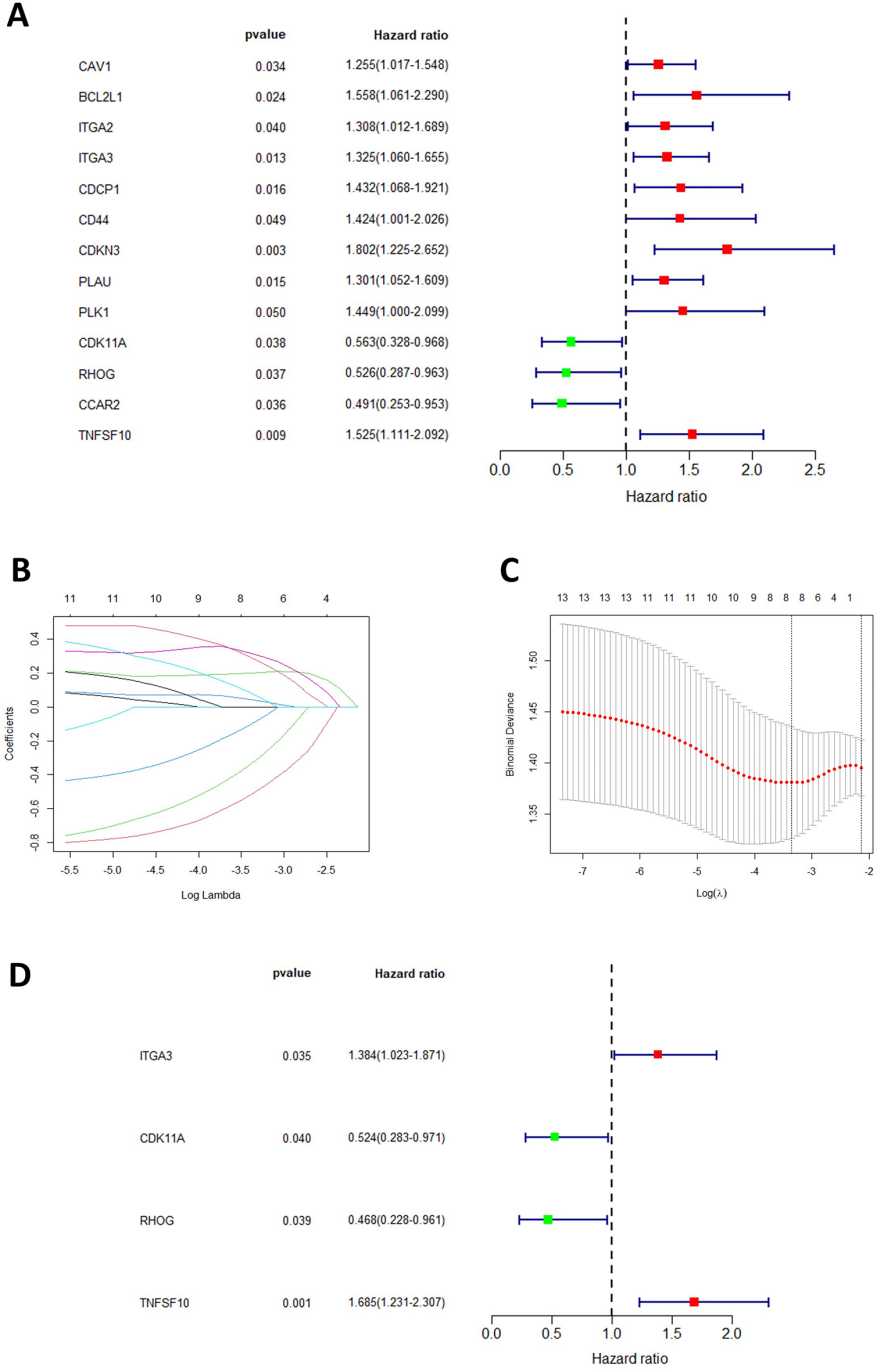
Cox-Lasso regression analysis to screen prognostic model genes. (**A**) A total of 13 nest loss apoptosis genes were considered to be associated with patient prognosis as a result of the results obtained after one-way Cox regression treating 55 target genes. (**B**) and (**C**) To prevent overfitting and enhance the generalization of the model, Lasso regression analysis was performed to obtain regression coefficient path plots and cross-validation plots, respectively. (**D**) Identification of final target genes by multifactorial Cox regression analysis.

**Figure 5:**
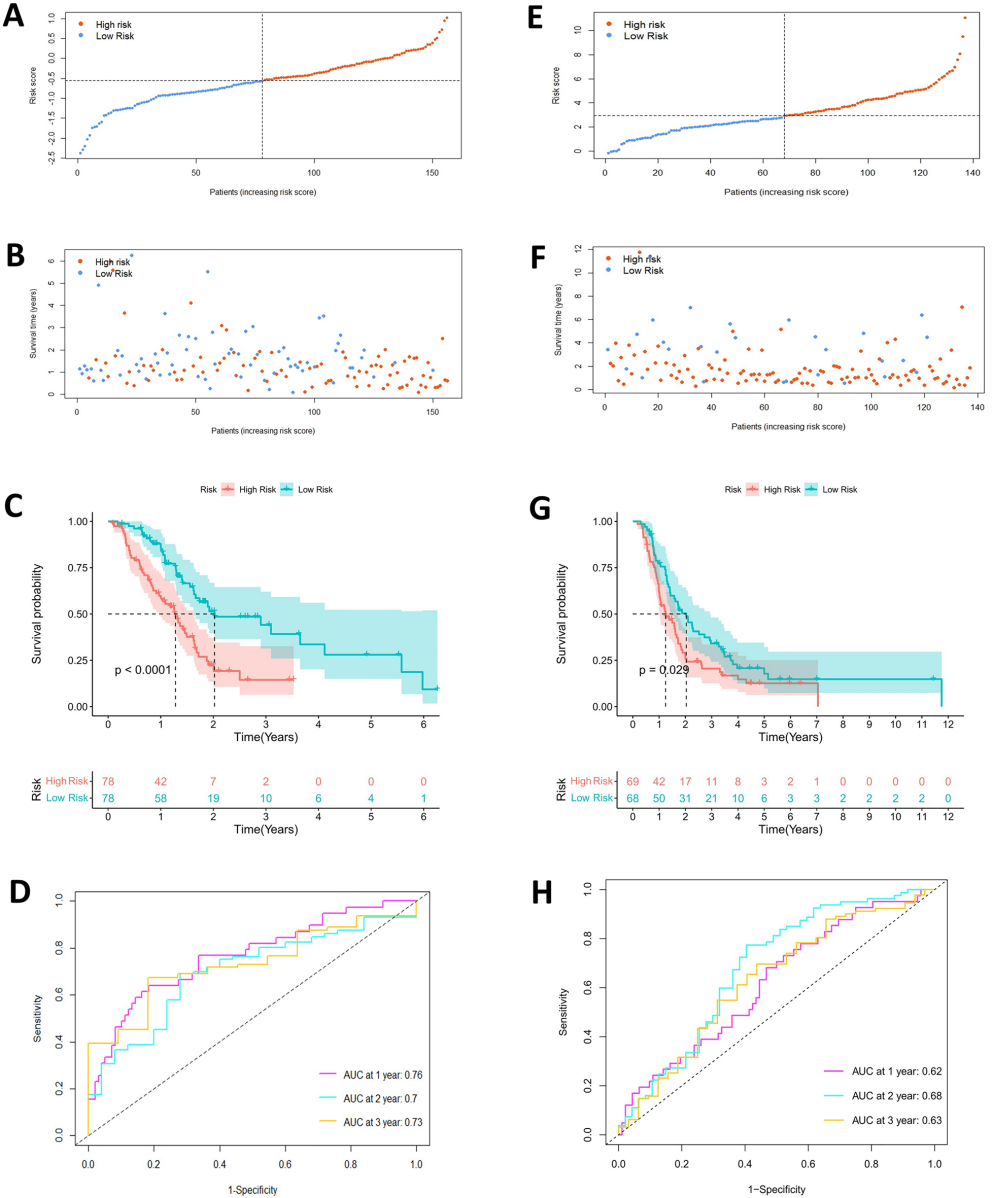
Construction of the prognostic model and external validation of the model. (**A**) Median risk score dividing patients in the high and low risk groups of the TCGA database sample. (**B**) Scatterplot of survival status in the high- and low-risk groups of the TCGA cohort. (**C**) Kaplan–Meier survival curves of patients in the high- and low-risk groups of the TCGA cohort, with color coverage of survival confidence intervals. (**D**) ROC curves of survival time at years 1–3 for the patients in the TCGA cohort (the horizontal coordinates represent the model specificity, and the vertical coordinates represent the sensitivity of the model). (**E**–**H**) Risk score calculation, survival status analysis, K-M survival curve plotting, and visualization of ROC curves at years 1, 2, and 3 for patients in the ICGC cohort using the same risk-prognostic model as the TCGA, yielding the same results as the TCGA cohort.

The univariate COX regression analysis (Fig. 4A) identified 13 genes with prognostic relevance (*P* < 0.05). To improve the model’s generalizability and prevent overfitting, lasso regression analysis (Fig. 4B-C) was conducted on these 13 genes, resulting in the identification of 8 prognostically relevant genes. Subsequently, a multifactorial COX regression analysis (Fig. 4D) was performed, leading to the identification of four prognosis-related genes (*P* < 0.05): ITGA3, CDK11A, RHOG, and TNFSF10. In the model, ITGA3 and TNFSF10 were identified as risk factors, with high expression adversely affecting the prognosis of PC patients, while CDK11A and RHOG were protective factors, with high expression being favorable for tumor prognosis. Using the established risk score formula, risk scores were computed for the tumor samples within the TCGA database. These samples were then categorized into two distinct risk groups based on the median score (Fig. 5A). A scatterplot was generated to display the survival rates of patients across these subgroups (Fig. 5B). The Kaplan–Meier curve demonstrated that patients in the high-risk category faced poorer outcomes compared to those in the low-risk group (Fig. 5C). The effectiveness of the model was further corroborated by the time-dependent ROC curve (Fig. 5D), exhibiting areas under the curve of 0.76, 0.7, and 0.73 for the first three years, respectively, thus affirming the model’s considerable predictive accuracy.

### Validation of anoikis-related gene risk signature model and construction of nomograms

**Figure 6:**
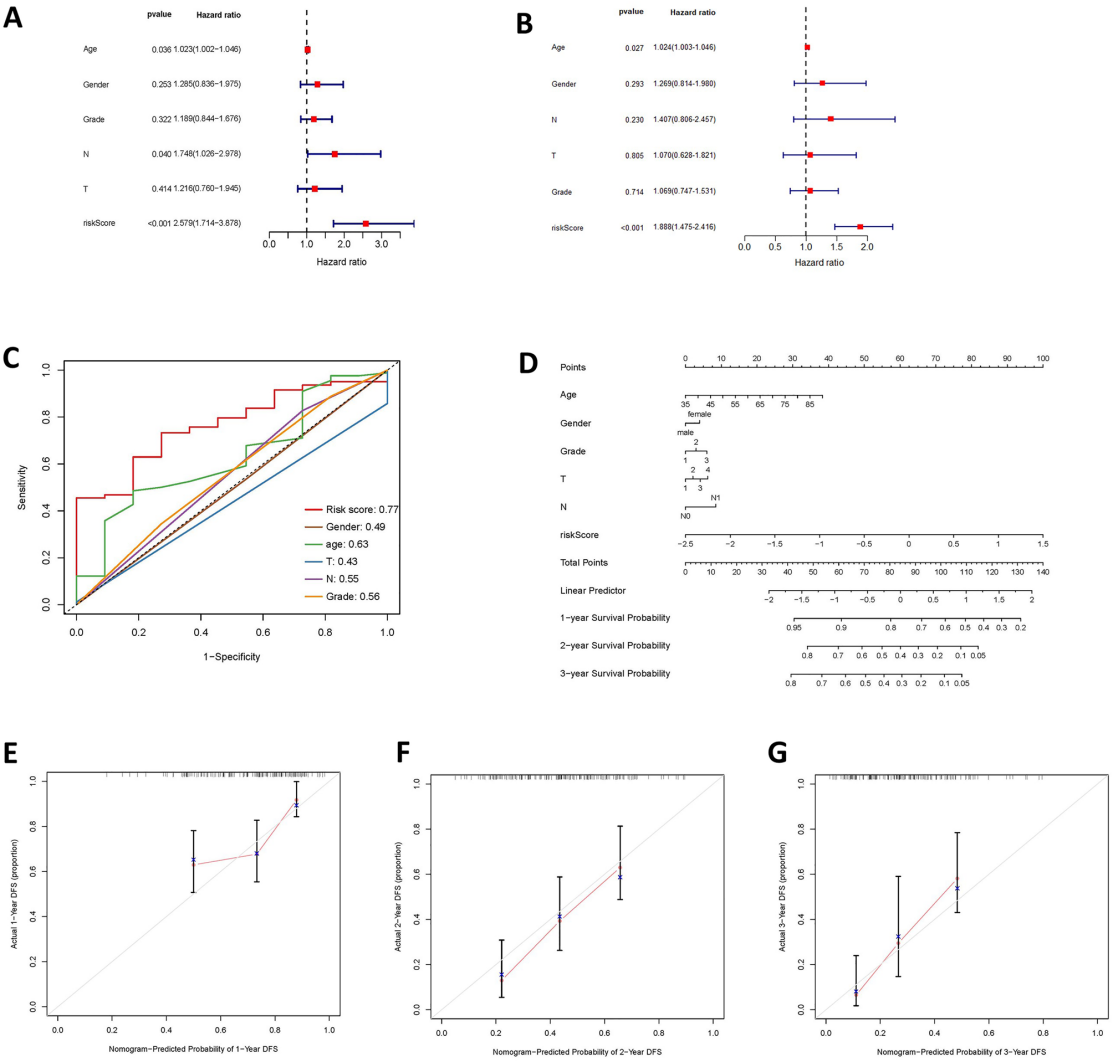
Performance evaluation of building Nomogram and prognostic models. (**A**) Results of one-factor Cox regression for clinical characteristics and risk characteristics of the TCGA cohort. (**B**) Results of multifactor Cox regression for clinical characteristics and risk characteristics of the TCGA cohort. (**C**) ROC curves of clinical characteristics and risk characteristics of the TCGA cohort. (**D**) Nomogram the survival rates of patients with PC at years 1, 2, and 3, constructed on the basis of the clinical characteristics and the risk scores. (**E**–**G**) Agreement between predicted and true outcomes at years 1–3 of the prognostic model.

The model’s validation was carried out using the ICGC database. Utilizing the risk scoring criteria established for the model, the samples in the ICGC database were classified into high-risk and low-risk groups (Fig. 5E). Scatter plots illustrated the survival status of the two risk subgroups (Fig. 5F). Kaplan–Meier charts, based on the ICGC data, mirrored the findings seen in the TCGA cohort. These results indicated that individuals categorized as high-risk had less favorable prognoses compared to those in the low-risk group (Fig. 5G). The time-dependent ROC curves for 1–3 years all exceeded 0.6, suggesting that the model has a high level of accuracy (Fig. 5H). The research further investigated the link between clinical attributes and the risk score of PAAD patients within the TCGA group, along with their prognostic outcomes. Both univariate and multivariate Cox regression analyses demonstrated that the risk score serves as an independent prognostic indicator for PAAD patients (*P* < 0.001) (Fig. 6A-B). Additionally, the risk score ROC curve showed promising results (Fig. 6C). Subsequently, nomograms were developed to predict patient prognosis based on the aforementioned model (Fig. 6D), and calibration curves were used to validate the nomogram predictions (Fig. 6E-G).

### Mutation status analysis and risk profile-based tumor mutation load analysis

**Figure 7:**
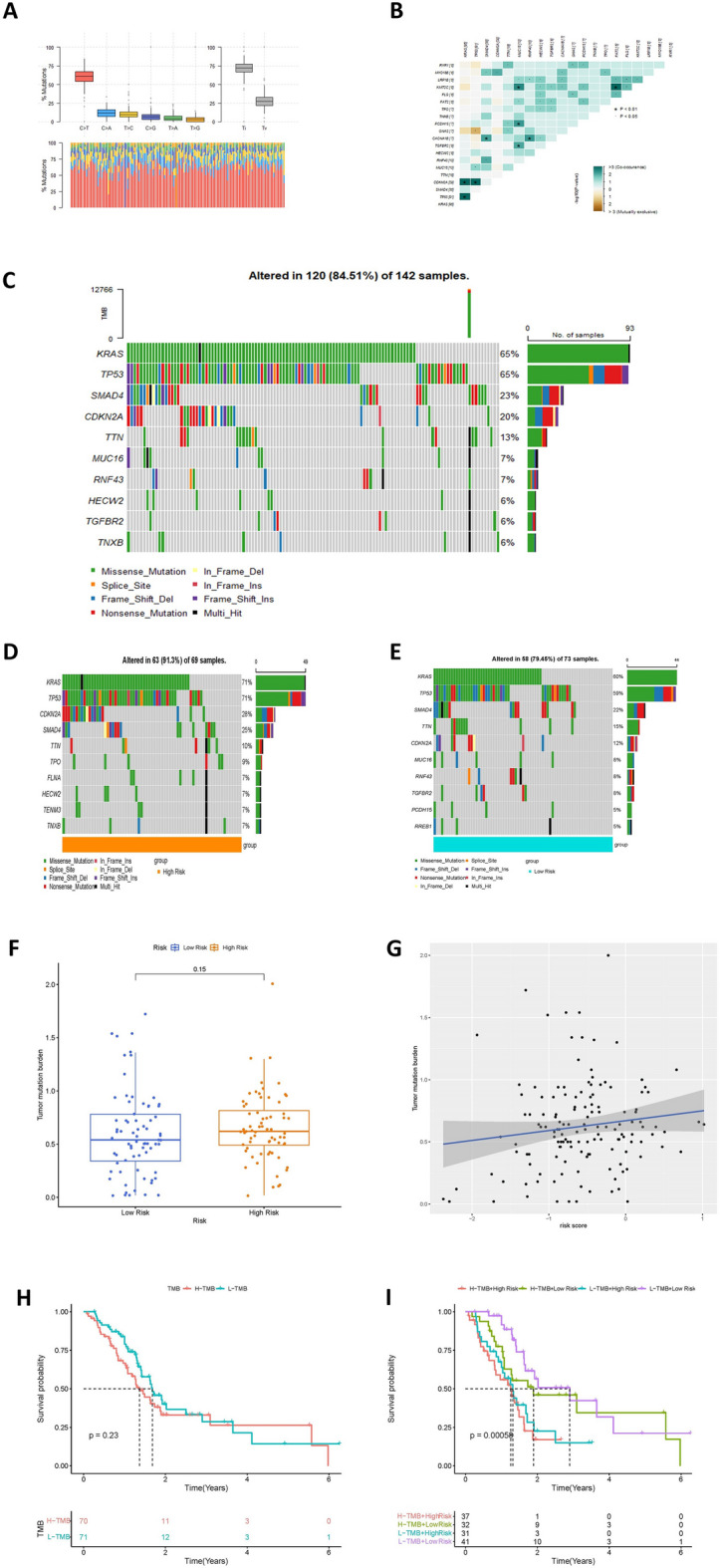
Mutation status and survival analysis of tumor samples in TCGA database. (**A**) Base mutation information in TCGA samples. (**B**) Correlation heatmap based on the top 20 mutated genes in TCGA samples. (**C**) Waterfall map of mutations in all samples of the TCGA cohort. (**D**-**E**) Waterfall map of mutations in the high-risk and low-risk groups in the TCGA cohort, with the high-risk group having a higher mutation rate. (**F**) Difference in TMB between high and low risk groups in the TCGA cohort. (**G**) Scatterplot of the correlation between TMB and risk scores. (**H**) Survival Kaplan–Meier curves between patients in the high mutation group (H-TMB) and the low mutation group (L-TMB). (**I**) Kaplan–Meier survival of the TCGA cohort for the combination of the risk profile score and the TMB score curves.

Analysis of the mutational status of samples from the TCGA cohort revealed that Ti mutations were more frequent than Tv mutations and were predominantly found in mutual substitution between cytosine and thymine, as depicted in the mutational heatmap (Fig. 7A). A correlation heatmap based on the top 20 mutated genes indicated a significant correlation between TP53, KRAS and CDKN2A (Fig. 7B). A waterfall plot displaying tumor mutations in all TCGA cohort samples highlighted the top 10 genes with the highest mutation percentages, with KRAS and TP53 being the top two genes (Fig. 7C). Waterfall charts were created for each risk category, showing a reduced mutation rate in the low-risk group compared to the high-risk group (Fig. 7D-E). The difference in TMB values between the two risk groups was found to be not statistically significant by plotting box plots, but scatter plots showed a positive correlation between risk scores and TMB values (Fig. 7F-G). Moreover, Kaplan–Meier curves indicated no substantial survival disparities between the high and low TMB groups (Fig. 7H). However, when combining the high and low-risk categories, it became apparent that patients in the high-risk and high-mutation subgroup faced significantly poorer prognoses compared to other groups (Fig. 7I).

### Evaluation of the tumor immune microenvironment in relation to the risk signature

**Figure 8:**
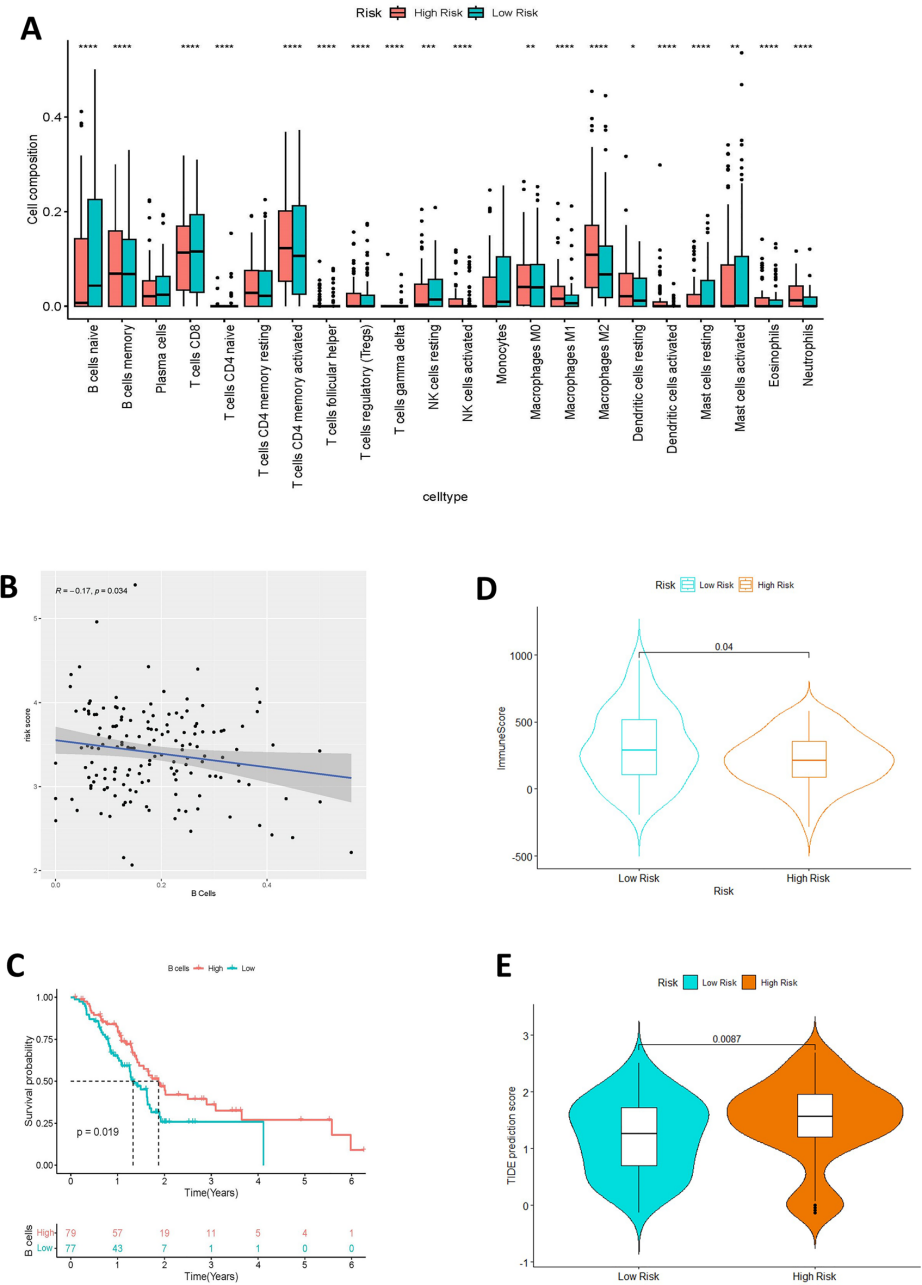
Immune infiltration analysis and immune-related scores based on risk score groupings. (**A**) Informative boxplot of immune cell composition in the TCGA cohort. (**B**-**C**) Dot plot of correlation analysis between risk scores and degree of B-cell infiltration and survival curves based on B-cell grouping. (**D**) Comparative violin plots of immune scores between high- and low-risk groups. (**E**) Tumor Immunity Dysfunction (TIDE) scores between the two risk groups in the TCGA cohort, with the high-risk group at higher risk for immune escape.

The box plots, illustrating immune infiltration according to the risk profile, showed variations in the immune microenvironment of the tumor across the two subgroups. Notably, there were more pronounced variances in B cells, M1 macrophages, and M2 macrophages (Fig. 8A). As the risk score increased, there was a corresponding reduction in B-cell infiltration (Fig. 8B). Kaplan–Meier survival curves based on B-cell subgroups indicated that subgroups with high infiltration of B cells exhibited a notably better prognosis (Fig. 8C). The high-risk group displayed lower immunity scores, suggesting reduced levels of immune cells (Fig. 8D). The violin plots demonstrated that the group with higher risk exhibited elevated TIDE scores, suggesting an increased probability of immune evasion and enhanced uresistance to immunotherapy (Fig. 8E).

### Anoikis-related gene mRNA expression in PC

**Figure 9:**
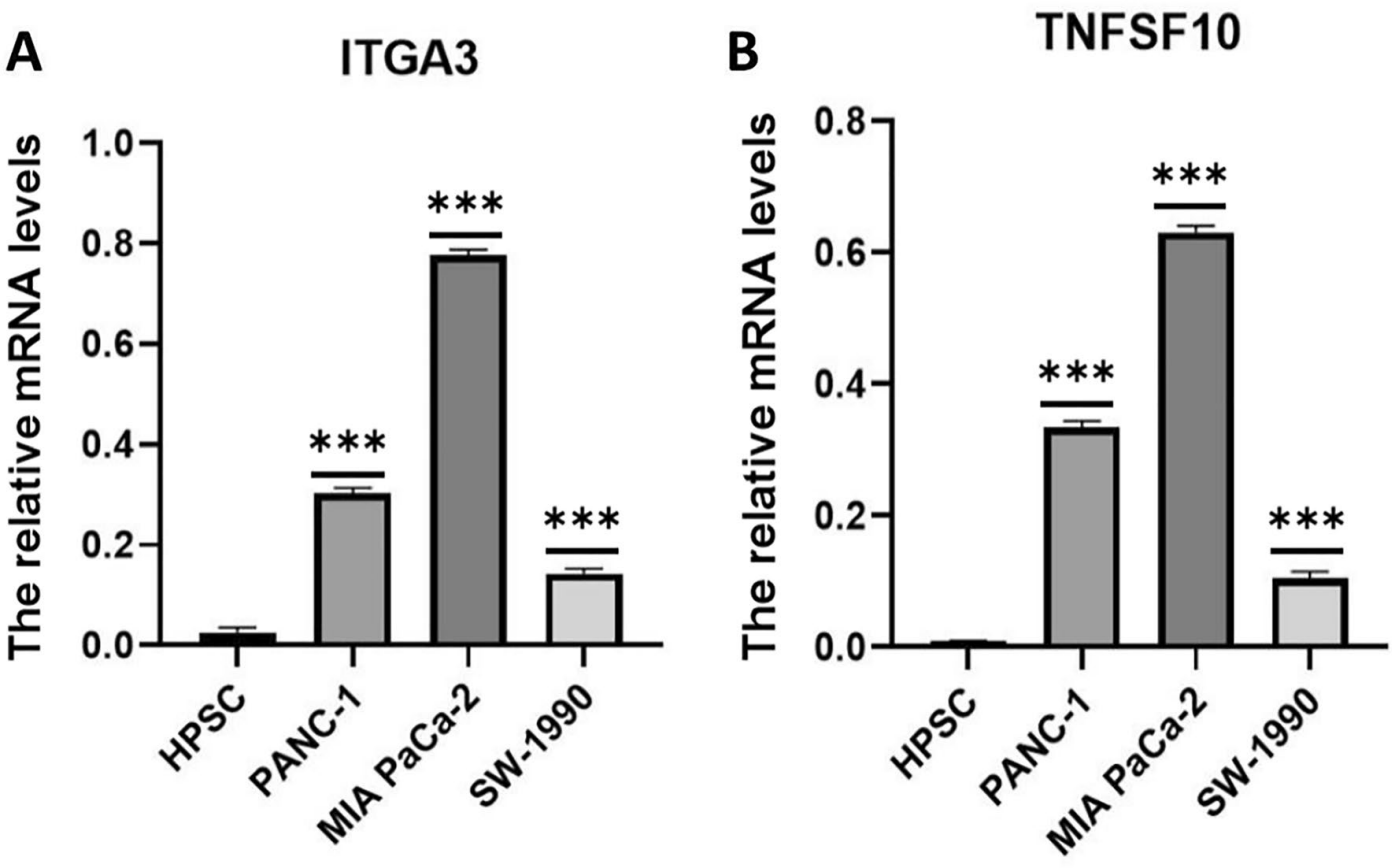
Expression of prognosis-related genes in cell lines. (**A**-**B**) Expression of ITGA3 and TNFSF10 in normal pancreatic cell line HPSC and three PC cell lines PANC-1, MIA PaCa-2, and SW-1990, with higher gene expression in all three tumor cell lines. ***P < 0.001.

To compare the variations in anoikis-related genes between normal pancreatic cells and tumor cells, we examined their expression in both types of cells. We found that two important apoptosis genes, ITGA3 and TNFSF10, were significantly more highly expressed in the PC tumor cells (panc1, miapaca2, and sw1990) compared to normal pancreatic cells. This finding aligns with the poor prognosis associated with PC patients (Fig. 9).

## Discussion

PC plays a major role in cancer-related mortality, with pancreatic ductal adenocarcinoma being the most common form of this disease. Known for its aggressive nature and resistance to chemotherapy, pancreatic ductal adenocarcinoma surpasses many other types of cancer in these aspects. The global incidence of pancreatic ductal adenocarcinoma has doubled in the last 25 years, and approximately half of newly diagnosed patients have metastatic PC^26^. Anoikis is a natural cell death process that helps regulate tissue balance and healthy growth. Disruption of this process can lead to the uncontrolled growth of abnormal cells in a suspended state^27^. Research has indicated that cancer cells have various ways to evade anoikis, which helps them survive and spread, ultimately contributing to the progression and metastasis of cancer^28^. Several articles have discussed the significant role of anoikis in the spread of malignant tumors like lung cancer and breast cancer^29,30^. Fewer connections have been examined between anoikis and the severity of tumors, immune infiltration, and tumor mutations in PC. This study leveraged transcriptomic data to explore the role of anoikis in PC, examine its impact on the tumor microenvironment, and predict the outcomes for patients with PC.

In our research, due to the limited number of normal samples in the TCGA database, the TCGA and GTEx data were merged, and outliers in the TCGA group were eliminated using a function in the WGCNA package to provide a more accurate representation of data variances. Cell adhesion and tumorigenesis-related pathways were examined through GO and KEGG enrichment, considering the close connection between anoikis and tumors. Subsequently, four prognostic genes associated with anoikis (ITGA3, CDK11A, RHOG, and TNFSF10) were identified using Cox and lasso regression, and a prognostic model was developed using these genes. Patients with elevated levels of ITGA3 and TNFSF10 exhibited poorer prognoses, whereas CDK11A and RHOG were identified as protective factors. The model classified patients into high-risk and low-risk categories, and subsequent survival analyses indicated that individuals in the high-risk group had shorter survival durations compared to those in the low-risk group. These findings were validated using the ICGC database. Cox regression analyses were also conducted on the clinical and risk characteristics of the patients but were not included in the analysis due to missing data from the sample of patients with tumor M stage. However, both T and N stages indicated a trend towards a poorer prognosis. The results indicated that the “risk score” was a stronger independent predictor of PC prognosis compared to other clinical factors. Moreover, the area under the ROC curve for the “risk score” exceeded 0.7, signifying its significant predictive value in assessing cancer patient outcomes.

The integrin alpha-3, produced by the ITGA3 gene, is a member of the integrin family. It is located on the cell membrane and functions as a cell adhesion molecule at the cell’s surface^31^. Existing studies have shown that ITGA3 is highly expressed in a variety of cancers, including thyroid^32^, colorectal^33^, prostate^34^, head and neck^35^, intrahepatic cholangiocarcinoma^36^, and esophageal^37^. Prior research has also demonstrated that increased ITGA3 expression is linked to reduced overall survival and recurrence-free survival in PC^38^, this aligns with the proven findings of this study. TNFSF10, also called TRAIL, is a ligand that induces apoptosis and is associated with TNF. TRAIL can attach to four receptors located on the cell membrane (TRAIL-R1-4). When TRAIL binds to TRAIL-R1 and TRAIL-R2, it triggers the activation of the intracellular death domain (DD), leading to cellular apoptosis^39^. The distinctive function of TRAIL makes it a promising treatment for cancer, but its use in clinical settings has not produced the expected results^40^. Further research has shown that the interaction between TRAIL and its death receptor TRAIL-R2 (DR5) not only triggers external apoptotic pathways to induce cell death, but also contributes to non-apoptotic biological functions. It has been found that these non-apoptotic processes can facilitate tumor cell invasion and metastasis^41^, mutations in K-Ras that promote colorectal cancer enhance the non-apoptotic TRAIL pathway to drive tumor advancement and spread^42^. Recent animal studies have shown that TRAIL induces the spread of PC tumor cells to distant locations in living organisms^43^, this echoes the results of this study. ROGH is a member of the RHO family of small GTPases, playing a crucial role in regulating the equilibrium of T-cells.RhoG plays a part in the internalization of TCR at the immunological synapse (IS), a process crucial for effective T-cell activation. However, the current knowledge about this process is still insufficient^44^, CDK11A belongs to a group of protein kinases that encode serine/threonine proteins and are cleaved by cysteine asparaginase. It is suggested that CDK11A may be involved in the process of apoptosis. Currently, there is a lack of research indicating RHOG and CDK11A as elements enhancing the prognosis for individuals with PC.

The Ras gene is the most commonly altered gene in human cancers, with mutations present in about 30% of cases. Ras proteins, which are membrane-bound, have GTPase activity that stimulates cell proliferation and angiogenesis when activated by external signals and bound to GTP. Mutant Ras continuously activates this process, leading to increased downstream signaling and promoting tumorigenesis. Among the three closely related Ras isoforms (K-Ras, H-Ras, and N-Ras), mutations in K-Ras alone account for nearly half of the cases^45^. Significantly, the most commonly mutated gene among PC patients in the TCGA cohort in this study was KRAS. K-Ras mutations have been identified in various types of cancer, including both intrahepatic and extrahepatic cholangiocarcinomas. Intrahepatic cholangiocarcinomas make up approximately 10% to 20% of cases with a low to high prevalence of K-Ras mutations, while extrahepatic cholangiocarcinomas have a much higher rate of about 40% to 60% of cases^46^. Mutations in the K-Ras gene are detected in colorectal adenomas in individuals at an early age^47^. Mutations in K-Ras have been identified across a diverse range of cancers, including lung cancer^48^, gastric cancer^49^, and several hematologic malignancies such as multiple myeloma and primary plasma cell leukemia^50^. Over 90% of PC patients have K-Ras mutations, and those with these mutations tend to have increased mobility and aggressiveness in their PC^51^. Examination of tumor mutations in varying risk categories showed that KRAS mutations were more prevalent in the high-risk subgroup than in the low-risk one.This suggests that the high-risk group is more aggressive and less responsive to treatment, leading to a poorer prognosis. The study also indicates that PC mutation status primarily involves similar nucleotides (Ti > Tv), possibly due to the higher likelihood of methylated cytosine converting to thymine in the natural environment. Furthermore, the research identified a positive association between risk scores and TMB, despite the lack of a substantial difference in TMB across the high-risk and low-risk groups. The survival analysis indicated a tendency towards poorer survival in the group with high mutations. Moreover, when integrating risk and mutation subgroups, it was observed that patients categorized under high mutation and high risk had the most unfavorable prognosis. In contrast, individuals in the low mutation and low risk categories experienced the most favorable outcomes, further validating the precision of the prognostic model.

Immunotherapy is a key component of cancer treatment, with several types currently providing positive clinical outcomes for certain patients. Immune cell infiltration in the tumor microenvironment plays a vital role in tumor progression and has a significant impact on the prognosis of cancer. However, cancer cells have developed various strategies, such as impaired antigen presentation and increased negative regulatory pathways, to evade immune cell attacks, thereby reducing the efficacy of immunotherapy^52^.

The tumor microenvironment is infiltrated by two types of immune cells: those belonging to the adaptive immune system and those from the innate immune system. Among the innate immune cells, natural killer cells can either directly destroy tumor cells or collaborate with adaptive immune cells for their elimination.Research increasingly shows that B cells, a subset of adaptive immune cells, also contribute significantly to antitumor immunity and are associated with positive outcomes for patients^53,54^. The presence of a high TMB is correlated with a high tumor neoantigen load and is considered as a gross indicator of enhanced immunotherapy efficacy^55^. Studying the TIME is therefore essential to understanding the complex interactions between all the actors involved in cancer immune escape mechanisms and treatment resistance and to learning how to exploit the immune system against PC cells. Moreover, TAMs are able to influence the activity of cytidine deaminase, responsible for gemcitabine metabolization, conferring resistance to this drug^56^. The interaction between stromal cells and cancer cells can have a dramatic impact on tumor growth, proliferation and survival, as well as drug response and drug resistance. Therefore, analyzing and understanding the TME and TIME is crucial for developing effective therapeutic strategies. Myeloid cells represent a major component of stroma cells and the high number of TAMs seems to correlate with prognosis in PC patients^57,58^. TAMs can promote neo-angiogenesis, cancer cell proliferation, and metastasis through the release of cytokines, proteases, and growth factors^59,60^. Additionally, TAMs can influence the activity of cytidine deaminase, which is responsible for gemcitabine metabolization, thereby conferring resistance to this drug^56^. In TME, TAMs are differentiated into two subpopulations called “M1” and “M2”, which have opposite roles: M2 have mainly anti-inflammatory functions, while M1 exert anti-cancer effects through the release of pro-inflammatory cytokines^61,62^. Tumor-associated macrophages do not only exhibit M1 and M2 polarization, but typically display M2-like characteristics. Additionally, M2-polarized macrophages have the ability to stimulate the generation of polyamines and L-proline, which in turn facilitates tumor development^63^. Variations in immune cell composition were noted among different risk subgroups within the TCGA cohort. B-cells have been identified as correlating with an improved prognosis in patients categorized under the low-risk group. Conversely, individuals in the high-risk group exhibited a more significant occurrence of M2-polarized macrophages. Visual analysis of immune scores indicated increased infiltration of immune cells in the low-risk group, suggesting reduced tumor purity and aiding in their improved prognosis. Furthermore, TIDE scores suggested a greater likelihood of immune escape in the high-risk group, which may explain the lack of significant difference in tumor-specific killer CD8^+^ T cells between the two subgroups. These findings indicate that the prognostic model based on sc apoptosis developed in this study can predict patient response to immunotherapy and serve as a guide for immunotherapy in PC.

In summary, we developed a predictive model for PC using four genes associated with cell death. Despite employing sophisticated statistical methods, some biases and confounding factors were inevitable. Current research indicates that^6^ TRAIL therapy has not achieved significant efficacy in pancreatic cancer and may even promote tumor progression. This study can utilize genetic testing to identify and avoid administering TRAIL therapy to high-risk patient groups. Unfortunately, the non-apoptotic pathways involved in these tumors require further research to develop more precise treatment strategies. With the rapid advancement of precision medicine, we believe that a deeper understanding of anoikis-related mechanisms will be achieved, leading to substantial progress in tumor research. The combination of these advancements will result in a qualitative leap in treatment outcomes. Given the retrospective nature of the study, additional factors may need to be considered in future research. While comparing data from multiple public databases has its limitations, the TMB and immune infiltration analyses conducted on various platforms enhance the credibility of our findings. Furthermore, quantitative real-time PCR results indicated a correlation between high expression levels of certain genes and poorer patient prognosis. The survival analysis, tumor mutation burden analysis, immune infiltration analysis, and quantitative real-time PCR based on risk profiles collectively confirmed the reliability of our prognostic model. Finally, the relationship between personalized treatment for PC and patient prognosis and quality of life is complex and varied. In clinical practice, immunotherapy is a powerful treatment method, but it needs to be based on rigorous medical monitoring^64^ and combined with other supportive measures, such as nutritional support^65^, to maximize patient benefits.

## Conclusions

The clustering dendrogram in WGCNA was used to identify and remove outliers from the samples. The remaining samples underwent Cox and Lasso regressions to conduct more precise and efficient data analysis. Additionally, immune microenvironment and tumor mutation analyses were carried out based on the risk scores from the model. The constructed model was found to enhance the accuracy and effectiveness of predicting PC patient prognosis. Furthermore, the model provided valuable insights for TRAIL therapies for PC with limited efficacy. To summarize, our research has enhanced the understanding of the molecular dynamics associated with anoikis gene-related PAAD, introducing novel targets and avenues for its clinical management.

## Data Availability

The raw data used in this study came from public open data platforms. The pancreatic sample datasets were retrieved from the TCGA (http://portal.gdc.cancer.gov), GTEx (https://gtexportal.org/home/) and ICGC (https://dcc.icgc.org/) databases.
